# High-Risk Penicillin Reaction Flags in the Medical Record

**DOI:** 10.1001/jamanetworkopen.2025.49081

**Published:** 2025-12-19

**Authors:** Samantha Novotny, Lina Elmansy, Jason Kwah, Jane Liao, Katelyn H. Wong, Cosby A. Stone, Ami P. Belmont

**Affiliations:** 1 Department of Internal Medicine, Yale School of Medicine, New Haven, Connecticut; 2 Yale School of Medicine, New Haven, Connecticut; 3 Section of Rheumatology, Allergy, and Immunology, Department of Internal Medicine, Yale School of Medicine, New Haven, Connecticut; 4 Pediatric Pulmonology, Allergy, Immunology, and Sleep Medicine, Department of Pediatrics, Yale School of Medicine, New Haven, Connecticut; 5 Division of Allergy, Pulmonary, and Critical Care, Department of Medicine, Vanderbilt University Medical Center, Nashville, Tennessee

## Abstract

This cohort study of patients with allergist-documented penicillin allergy label assesses agreement between primary reaction symptoms documented by allergists and the allergy module of the electronic health record.

## Introduction

Evaluating penicillin allergy labels (PALs) is important to public health. PALs are common, largely inaccurate, and associated with preventable adverse outcomes due to inappropriate prescribing.^1^ Guidelines support classifying patients with PALs as being at high or low risk for true allergy.^1^ Patients with low-risk reaction histories can undergo direct penicillin challenges and receive other β-lactam antibiotics, including cephalosporins, without testing.^1^ Although prior research has shown that reaction descriptions in the allergy module of the electronic health record (AM-EHR) are incomplete and misclassify intolerances as allergy,^2,3^ the AM-EHR is frequently used to understand characteristics of reactions and reaction risk. We conducted this study to assess agreement among (1) penicillin reaction descriptions in the AM-EHR and allergist-obtained histories, (2) reactions marked as high risk in the AM-EHR and by allergists, and (3) reactions marked as high risk in the AM-EHR and penicillin allergy testing results.

## Methods

This retrospective cohort study, which received institutional review board exemption and a waiver of informed consent for secondary use of health information for research purposes, followed the Strengthening the Reporting of Observational Studies in Epidemiology (STROBE) reporting guideline. It included patients seen for consultative visits at a US university-affiliated allergy/immunology clinic from August 1, 2023, to April 30, 2024, who had an allergist-documented PAL. We recorded data in the Epic AM-EHR time marked before the visit. We assessed agreement between primary reaction symptoms documented by allergists and the AM-EHR. For patients with multiple reaction symptoms, the most severe symptom was used for comparison. We assessed agreement between allergists’ risk assessments and high-, moderate-, and low-risk flags in the AM-EHR.^4^ We performed descriptive analyses and calculated agreement through percentage agreement and the Cohen κ, with a 2-sided *P* < .05 considered statistically significant. Analyses were performed with Stata statistical software, version 18.0 (StataCorp). Additional information is available in the eMethods in Supplement 1.

## Results

Among 230 patients (median [IQR] age, 37 [30-60] years; 191 [83.0%] female), 224 [97.4%] had PALs in the AM-EHR (Table 1). Primary reaction symptoms identified in the AM-EHR aligned with allergists’ assessments in 50.9% (95% CI, 44.4% to 57.4%; κ = 0.36; 95% CI, 0.27 to 0.44) (Table 2). There was a higher percentage agreement for identifying cutaneous symptoms (eg, 74.7% [95% CI, 69.1% to 80.4%] agreement for any rash; κ = 0.39; 95% CI, 0.25 to 0.52).^5^ In comparison, there was only 41.7% (95% CI, 35.3% to 48.2%) agreement of AM-EHR reaction risk with allergists’ assessments (κ = 0.01; 95% CI, −0.04 to 0.06). Cross-tabulated data of additional high-risk symptoms (eg, anaphylaxis) demonstrated discordance between allergist and AM-EHR documentation (Table 2). Among 107 patients flagged as being at high risk by the AM-EHR, only 1 was deemed as being at high risk by an allergist. During the study period, 119 patients underwent penicillin allergy testing; 1 patient tested positive. After negative penicillin skin testing results, she underwent a 2-step amoxicillin challenge. She developed hives and nausea after the second dose (500 mg of amoxicillin). Her symptoms resolved with an epinephrine injection and famotidine. She had no high-risk flags in the AM-EHR. Among 118 patients who tested negative, 60 (50.8%) had high-risk flags in the AM-EHR.

**Table 1.** Baseline Patient Demographics and Medical History

Characteristic	No. (%) of patients (N = 230)
Demographic characteristics	
Age, median (IQR), y	37 (30-60)
Gender	
Female	191 (83.0)
Male	36 (15.7)
Other^a^	3 (1.3)
Race^b^	
Asian	4 (1.7)
Black or African American	32 (13.9)
White	172 (74.8)
Other or unknown^c^	22 (9.6)
Hispanic or Latinx ethnicity^b^	30 (13.0)
Primary language not English	7 (3.0)
Medical and allergic history^d^	
Charlson Comorbidity Index, median (IQR)	0 (0-2)
Referred to allergy clinic for penicillin allergy label	148 (64.3)
Pregnant at time of allergist visit	81 (35.2)
Asthma	70 (30.4)
Eczema	34 (14.8)
Allergic rhinoconjunctivitis	104 (45.2)
Chronic urticaria or angioedema	27 (11.7)
Food allergy	36 (15.7)
Venom allergy	11 (4.8)
Multiple drug allergy labels (≥2 allergy labels)	72 (31.3)
Penicillin allergy label included in allergy module of EHR	224 (97.4)
Penicillin index reaction history^e^	
Anaphylaxis	5 (2.2)
Angioedema	8 (3.5)
Nonurticarial rash	101 (43.9)
Urticarial rash	68 (29.6)
Remote (≥5 y earlier)	196 (85.2)
Penicillin allergy assessments^e^	
Low-risk reaction	200 (87.9)
Moderate-risk reaction	28 (12.2)
High-risk reaction	2 (0.87)

Abbreviation: EHR, electronic health record.

^a^ Other gender included nonbinary, transgender male, and transgender female.

^b^ Race and ethnicity were obtained from the EHR.

^c^ Other race referred to patients who reported that their race was not listed in the demographics module of the EHR.

^d^ History was obtained via medical record review of allergists’ notes, other practitioners’ notes, and the EHR problem list.

^e^ The index reaction was considered the initial reaction to a penicillin antibiotic. These rows describe reaction details documented by the allergists. Of note, explicit documented risk assessments were missing in 83 allergists’ notes (36%). A board-certified allergist used the well-validated clinical decision rule PEN-FAST to perform risk stratification on all patients, including patients who had missing risk stratification explicitly stated in the allergists’ notes.^4^ The PEN-FAST rule is used as standard of care at this academic center. Upon reviewing all records, 146 of 147 (99.3%) of the study allergists’ assessments agreed with risk assessments in the allergists’ initial clinical notes (see eMethods in Supplement 1 for additional information).

**Table 2.** Penicillin Allergy Reaction Symptoms and Reaction Risk Detailed by Allergists vs EHR Allergy Module

Allergy module of EHR	Allergist documentation			Agreement, % (95% CI)	κ value (95% CI)^a^
	Low	Moderate	High		
Primary reaction symptom	NA	NA	NA	50.9 (44.4 to 57.4)	0.36 (0.27 to 0.44)
Reaction risk^b^				NA	NA
Low	93	11	1	41.7 (35.3 to 48.2)	0.01 (−0.04 to 0.06)
Moderate	16	2	0		
High	91	15	1		
Any rash	Yes	No	NA	NA	NA
Yes	135	24	NA	74.7 (69.1 to 80.4)	0.39 (0.25 to 0.52)
No	34	37	NA		
Nonurticarial rash^c^	Yes	No	NA	NA	NA
Yes	48	29	NA	64.3 (58.1 to 70.6)	0.26 (0.13 to 0.38)
No	53	100	NA		
Urticarial rash^c^	Yes	No	NA	NA	NA
Yes	45	45	NA	70.4 (64.5 to 76.4)	0.35 (0.23 to 0.48)
No	23	117	NA		
Angioedema	Yes	No	NA	NA	NA
Yes	3	6	NA	95.2 (92.4 to 98.0)	0.33 (0.03 to 0.63)
No	5	216	NA		
Anaphylaxis	Yes	No	NA	NA	NA
Yes	2	8	NA	95.2 (92.4 to 98.0)	0.25 (−0.06 to 0.54)
No	3	217	NA		

Abbreviations: EHR, electronic health record; NA, not applicable.

^a^ The following Cohen κ cutoff criteria were used: less than 0.00 indicates poor agreement, 0.00 to 0.20 indicates slight agreement, 0.21 to 0.40 indicates fair agreement, 0.41 to 0.60 indicates moderate agreement, 0.61 to 0.80 indicates substantial agreement, and 0.81 to 1.00 indicates almost perfect agreement.^5^

^b^ Reaction severity flags in the AM-EHR were compared with allergist documentation of reaction risk. Additional details are available in the eMethods in Supplement 1.

^c^ Sensitivity analyses of patients presenting with any rash analyzed by urticarial and nonurticarial rashes.

## Discussion

There is public health benefit to determining evidence-based strategies to address PALs efficiently, accurately, and reliably. This study compared details in the AM-EHR, including high-risk flags, with allergists’ assessments and penicillin allergy testing results. AM-EHR reaction descriptions were inconsistent with allergist-obtained histories in more than half of cases. Furthermore, although approximately 50% of patients had high-risk flags in the AM-EHR, this rarely aligned with allergists’ assessments and never aligned with testing results.

Our results must be interpreted within the context of several limitations, including the single-center retrospective design and predominantly female and White patient sample, which included pregnant patients. Additionally, time between AM-EHR documentation and allergist consultation was unavailable. Low prevalence for select variables may have impacted κ values.^5^ Nonetheless, this study is strengthened by the use of allergist assessments and testing. It builds upon prior findings showing that the AM-EHR is often incomplete or inconsistent with patient-reported history and misclassifies intolerances as allergies.^2,3,6^

Our findings suggest that details in the AM-EHR and high-risk flags regarding PALs are unreliable. This issue should be considered in both research studies and clinical practice. These findings reinforce the need for implementation studies, quality initiatives, education, and cross-specialty guidelines to improve AM-EHR documentation and clinical decision support for PALs.^6^

## Notes

Supplement 1. **eMethods.** Supplementary Methods
eReferences

Supplement 2.
Data Sharing Statement
