# Supplementary material for: High-Risk Penicillin Reaction Flags in the Medical Record
Source: JAMA Netw Open. 2025 Dec 19;8(12):e2549081. doi: 10.1001/jamanetworkopen.2025.49081 (PMC12717615; doi:10.1001/jamanetworkopen.2025.49081)
Supplement: Supplement 1. — eMethods. Supplementary Methods eReferences [file jamanetwopen-e2549081-s001.pdf]

Supplemental Online Content

Novotny S, Elmansy L, Kwah J, et al. High-risk penicillin reaction flags in the medical record. *JAMA Netw Open*. 2025;8(12):e2549081. doi:10.1001/jamanetworkopen.2025.49081

eMethods. Supplementary Methods
eReferences

This supplemental material has been provided by the authors to give readers additional information about their work.

© 2025 Novotny S et al. *JAMA Network Open*.

eMethods. Supplementary Methods

Data Collection and Coding

Overall and Sociodemographic Data Collection from the Electronic Health Record (EHR)

Data were collected from the Yale New Haven Epic™ electronic health record (EHR) and recorded in REDCap. Race and ethnicity data were included to understand the sociodemographic background of patients who had evaluation by an allergist. These data were obtained from the demographics section of the EHR.

Description of the Yale New Haven Hospital Epic™ Allergy Module

Allergy data can be input into the allergy module of the EHR by any healthcare worker (e.g., medical assistants, nurses, pharmacists, advanced care providers, physicians). The allergy module of the EHR has undergone revisions with Epic™ software updates over time. The following describes how allergy module input operates at the time of this publication. When creating a new allergy entry in the allergy module, healthcare workers are required to choose at least one symptom from a drop-down list. There is also an option to add additional details via free text. The drop-down list of symptoms includes common reaction symptoms (e.g., rash, urticaria, anaphylaxis, angioedema, diarrhea), as well as "other" and "unknown." Some of the symptoms on the drop-down list are automatically assigned "high", "medium", or "low" severity flags. For example, the following symptoms are assigned a high-severity flag automatically: anaphylaxis, angioedema, aplastic anemia, chest pain, hives, malignant hyperthermia, neuroleptic malignant syndrome, shortness of breath, Stevens-Johnson syndrome, and swelling. Reaction symptoms that trigger medium-severity flags include photosensitivity and "other." In contrast, the following symptoms are assigned a low-severity flag automatically: anxiety, dermatitis, diarrhea, itching, nausea, nausea and vomiting, palpitations, rash, or

© 2025 Novotny S et al. *JAMA Network Open*.

tinnitus. Healthcare workers can manually edit severity flags and can choose to manually add flags for other symptoms that do not have automatically assigned flags. Healthcare workers can also label the entry as an allergy, intolerance, or contraindication, although this is not required. Allergy module entries persist in the EHR unless manually updated or deleted by a healthcare worker. Deletion requires the healthcare workers to select a reason for deletion from a drop-down list with the option to add additional comments. Deleted allergy entries can be seen when reviewing the full allergy module (depending on user preferences), but are not visualized as active allergies in the EHR. Of note, the Yale New Haven Health system switched to Epic™ in 2013. Allergy records from the former EHR were carried over into the Yale New Haven Health Epic™ EHR automatically at that time, and there is often not a date of initial entry noted from the prior EHR. We recorded data in the Epic™ allergy module time-marked *before* the initial visit in the Allergy/Immunology clinic.

Data Collection and Coding

We recorded the allergists' and allergy module of the EHR (AM-EHR) descriptions of index reactions to penicillin antibiotics. The index reaction was defined as the initial reaction to a penicillin antibiotic. These data were used to assess percentage agreement between reaction descriptions, referred to as the primary symptom, as well as agreement between reporting of individual symptoms (e.g., urticaria, angioedema, anaphylaxis). When ascertaining agreement between primary reaction symptoms, if more than one symptom was reported, we used the most severe symptom (e.g., most indicative of a severe allergic reaction). We used the following symptom severity scale, listed from highest to lowest severity: severe cutaneous adverse reaction (e.g., blistering rashes, Stevens-Johnson syndrome), anaphylaxis, angioedema/other swelling, shortness of breath, urticaria/hives, other non-urticarial rash, gastrointestinal symptoms (e.g., abdominal pain, nausea, emesis, diarrhea), non-allergic symptoms (e.g., headache, anxiety, hallucinations), family history of reaction, and unknown reaction. Reported

© 2025 Novotny S et al. *JAMA Network Open*.

anaphylaxis and angioedema was only recorded if there was explicit documentation of suspicion for anaphylaxis (or angioedema) by the allergist or in the AM-EHR. Coding was done by consensus by two independent reviewers, one of whom is a board-certified internist and allergist/immunologist. Any discrepancies in coding were resolved after discussion. Lastly, we also recorded penicillin allergy testing results.

High-, medium-, and low-severity flags in the allergy module were used for comparison with allergists' assessments of high-, moderate-, and low-risk reactions, respectively. If the AM-EHR did not have any flag, this was coded with low-severity flags (because it would be treated as such clinically). We recorded the allergists' overall risk assessment. It is standard of care in our academic center clinic to use the PEN-FAST clinical decision rule for risk assessment.[1] When a risk assessment was not documented, two study team members, including a board-certified internist and allergist/immunologist adjudicated risk using the PEN-FAST clinical decision rule and reached consensus.[1] When a reaction was listed as "unknown" this was considered low-risk, consistent with the standard of care and risk adjudication with the PEN-FAST clinical decision rule.[1,2] The study had set an initial pre-determined criteria that if the study reviewers' risk assessment for all patients using the same protocol had greater than 95% agreement with allergist documented risk assessments, it would be acceptable for the study team members to risk stratify any patients who did not have explicitly stated risk assessments (but whose notes contained the relevant details to perform risk stratification with PEN-FAST). These results are reported in Table 1.

Penicillin testing was performed in alignment with current drug allergy guidelines.[2] Patients underwent penicillin skin testing followed by an amoxicillin challenge, or a direct amoxicillin challenge (with no preceding skin testing).[2] The testing strategy was determined by the treating allergist at an initial consultative visit. Skin testing included skin prick testing and intradermal

© 2025 Novotny S et al. *JAMA Network Open*.

testing with PRE-PEN® (benzylpenicilloyl polylysine, 6.0×10^{-5} M), penicillin G (10,000 units/mL), and ampicillin (10 mg/mL) in duplicate. Amoxicillin challenges were completed using a two-step challenge protocol (e.g., 50 mg amoxicillin, followed by a 30 minute observation window, then 450 mg amoxicillin followed by a 60 minute observation window). All patients were evaluated by board-certified or eligible allergists.

Data Analysis

We performed descriptive analyses. We also calculated agreement through both percentage agreement and Cohen's kappa, with a 2-sided p-value <0.05 considered statistically significant.

Percentage agreement was defined as the total number of agreeing responses for a particular variable divided by the total number of responses. Interpretation of the kappa statistic was based on the following delineations as proposed by Landis and Koch (1977): κ <0.00, poor agreement; κ 0.00-0.20, slight agreement; κ 0.21-0.40, fair agreement; κ 0.41-0.60, moderate agreement; κ 0.61-0.80, substantial agreement; κ 0.81-1.00, almost perfect agreement.[3] Of note, for the analysis assessing agreement between primary symptoms, missing data were included in the data analysis (coded as "other" or "unknown"). Data analysis was performed using STATA 18.0 (StataCorp, College Station, Texas). The Cohen kappa statistic and its confidence intervals were calculated with the STATA kappaetc package.[4-6]

eReferences (see full reference list in main text)

© 2025 Novotny S et al. *JAMA Network Open*.

1. Trubiano JA, Vogrin S, Chua KYL, et al. Development and Validation of a Penicillin Allergy Clinical Decision Rule. *JAMA Intern Med*. 2020;180(5):745-752.
2. Khan DA, Banerji A, Blumenthal KG, et al. Drug allergy: A 2022 practice parameter update. *J Allergy Clin Immunol*. 2022;150(6):1333-1393.
3. Landis JR, Koch GG. The Measurement of Observer Agreement for Categorical Data. *Biometrics*. 1977;33(1):159-174.
4. Cohen J. A Coefficient of Agreement for Nominal Scales. *Educational and Psychological Measurement*. 1960;20(1):37-46.
5. Cohen J. Weighted kappa: Nominal scale agreement provision for scaled disagreement or partial credit. *Psychological Bulletin*. 1968;70(4):213-220.
6. Klein D. KAPPAETC: Stata module to evaluate interrater agreement. *Statistical Software Components S458283, Boston College Department of Economics*. 2016, revised 11 Aug 2022.

© 2025 Novotny S et al. *JAMA Network Open*.
