# Supplementary material for: High-Risk Penicillin Reaction Flags in the Medical Record
Source: JAMA Netw Open. 2025 Dec 19;8(12):e2549081. doi: 10.1001/jamanetworkopen.2025.49081 (PMC12717615; doi:10.1001/jamanetworkopen.2025.49081)
Supplement: Supplement 2. — Data Sharing Statement [file jamanetwopen-e2549081-s002.pdf]

Data Sharing Statement

Novotny. High-Risk Penicillin Reaction Flags in the Medical Record. *JAMA Netw Open*. Published December 19, 2025. doi:10.1001/jamanetworkopen.2025.49081

Data
Data available: Yes
Data types: Deidentified participant data, Data dictionary
How to access data: Data will be made available upon request to corresponding author.
When available: With publication

Supporting Documents
Document types: Statistical/analytic code
How to access documents: This will be available upon request to corresponding author.
When available: With publication

Additional Information
Who can access the data: Researchers with proposed use of the data.
Types of analyses: It will be available for any purpose.
Mechanisms of data availability: With signed data access agreement.
